# Unraveling the Cross-Tissue Neuroimmune–Vascular Genetic Architecture of Migraine Using Integrated Multi-Omics, Single-Cell, and Spatial Transcriptomics: Prioritizing T-Cell Regulatory Networks and Peripheral Targets

**DOI:** 10.3390/ijms27031615

**Published:** 2026-02-06

**Authors:** Chung-Chih Liao, Ke-Ru Liao, Jung-Miao Li

**Affiliations:** 1 School of Medicine, Chung Shan Medical University, Taichung 40201, Taiwan; cshy2232@csh.org.tw; 2 Department of Integrated Chinese and Western Medicine, Chung Shan Medical University Hospital, Taichung 40201, Taiwan; 3 Department of Neurology, Yuanlin Christian Hospital, Yuanlin 51052, Taiwan; u9401407@cmu.edu.tw; 4 School of Chinese Medicine, College of Chinese Medicine, China Medical University, Taichung 40402, Taiwan; 5 Department of Chinese Medicine, China Medical University Hospital, Taichung 40447, Taiwan

**Keywords:** migraine, neuroimmune–vascular axis, functional genomics, single-cell RNA sequencing, spatial transcriptomics, PTK2B

## Abstract

Migraine is a complex neurovascular disorder in which immune signaling intersects with vascular and neural circuits, yet the tissue and cell-type context of common genetic risk remains incompletely defined. We integrated large-scale migraine genome-wide association study (GWAS) summary statistics with Genotype-Tissue Expression (GTEx) v8 expression and splicing quantitative trait loci (eQTLs and sQTLs), Bayesian co-localization, single-cell RNA sequencing of peripheral blood mononuclear cells (PBMCs) from migraine cases and controls, a healthy single-cell multi-omics atlas (assay for transposase-accessible chromatin (ATAC) plus RNA), high-dimensional weighted gene co-expression network analysis (hdWGCNA), and embryo-level spatial transcriptomics. Genetic signals were enriched in peripheral arteries, heart, and blood, and gene-level enrichment highlighted mucosal–smooth muscle organs including the bladder and the cervix endocervix. Cell-type prioritization consistently implicated endothelial and vascular smooth muscle lineages, with additional support for inhibitory interneurons and bladder epithelium. In PBMC T cells, co-expression modules capturing cytotoxic/activation and T-cell receptor signaling programs contained migraine-prioritized genes, including PTK2B, nominating immune activation circuitry as a component of genetic susceptibility. Spatial projection further localized risk concordance to craniofacial/meningeal interfaces and visceral smooth muscle–mucosal structures. Together, these analyses delineate a systemic neuroimmune–vascular architecture for migraine and provide genetically anchored candidate pathways and targets for mechanistic and therapeutic follow-up.

## 1. Introduction

Migraine is a highly prevalent and disabling primary headache disorder that imposes a substantial burden on individuals and health systems worldwide. Analyses from the Global Burden of Disease (GBD) 2019 study confirmed migraine as the second leading cause of years lived with disability globally and the leading cause among young and middle-aged women [1]. Using GBD 2021 data, more recent assessments estimate that approximately 1.16–1.20 billion people—around one-seventh of the world’s population—were living with migraine in 2021, with the highest age-standardized rates in high-sociodemographic-index regions and a disproportionate burden among women and working-age adults [2,3]. Over the past three decades, global migraine prevalence and migraine-attributable disability-adjusted life years have risen by more than 50%, indicating that demographic expansion and changing exposures have outpaced therapeutic gains [3,4]. Cost-of-illness studies further show that migraine is associated with high direct health-care expenditures and substantial productivity losses, leading to an annual economic burden in the United States that is conservatively estimated in the tens of billions of US dollars [5,6]. Together, these observations underscore the need for deeper mechanistic insights to support more effective prevention and treatment strategies [7].

Current models view migraine as a complex disorder arising from the interaction of neuronal hyperexcitability, activation of the trigeminovascular system, neurogenic inflammation, and context-dependent vascular changes, with cortical spreading depression (CSD) acting as a key initiator or amplifier in migraine with aura [8,9]. Across the premonitory and headache phases, hypothalamic activation preceding pain onset, release of calcitonin gene-related peptide (CGRP) and other neuropeptides from trigeminal afferents, meningeal neurovascular–neuroimmune crosstalk, and subsequent central sensitization together shape the characteristic unilateral throbbing pain, sensory hypersensitivity, and autonomic symptoms [9,10,11]. Marked clinical heterogeneity—between migraine with and without aura and among patients with prominent autonomic, immune-linked, or affective features—supports conceptualizing migraine as a multisystem brain disorder that variably recruits central nociceptive circuits and peripheral sensory–autonomic inputs [12,13].

In recent years, with the advancement of the Human Genome Project and the construction of large-scale biological databases, our understanding of the genetic architecture of migraine has made significant progress [14,15]. However, research on how migraine operates through expression quantitative trait loci (eQTLs) and splicing quantitative trait loci (sQTLs) in human tissues, as well as through the regulation of specific cell types, gene expression networks, and signaling pathways, remains a weak link in current studies. Traditional single-level research methods are inadequate for systematically revealing its multi-scale mechanisms; thus, there is an urgent need to integrate genetic data with multi-omics functional data.

To systematically dissect the complex mechanisms of migraine, this study is based on large-scale population genetic summary data, integrating multi-level data such as single-cell RNA sequencing (scRNA-seq), spatial transcriptomics, and single-cell-level chromatin accessibility information, employing cutting-edge computational methods like cross-omics integration analysis to comprehensively analyze the biological basis of migraine in systemic, cellular, and molecular dimensions. Specifically, we aim not only to identify risk gene loci, validate their tissue association information, and reveal their regulatory effects in different cell types but also to focus on the specific roles of candidate pathways such as regulation in the overlapping mechanisms, as well as the spatial expression distribution of risk genes at the embryonic level. This study seeks to uncover the key molecules mediating migraine and their functional connections, thereby providing solid theoretical foundations and data support for future targeted therapies, biomarker discovery, and early identification of high-risk populations. Ultimately, we hope these findings can offer new perspectives and strategies for unraveling migraine.

## 2. Results

### 2.1. Enrichment of Migraine Associations in eQTLs and sQTLs

**Figure 1:**
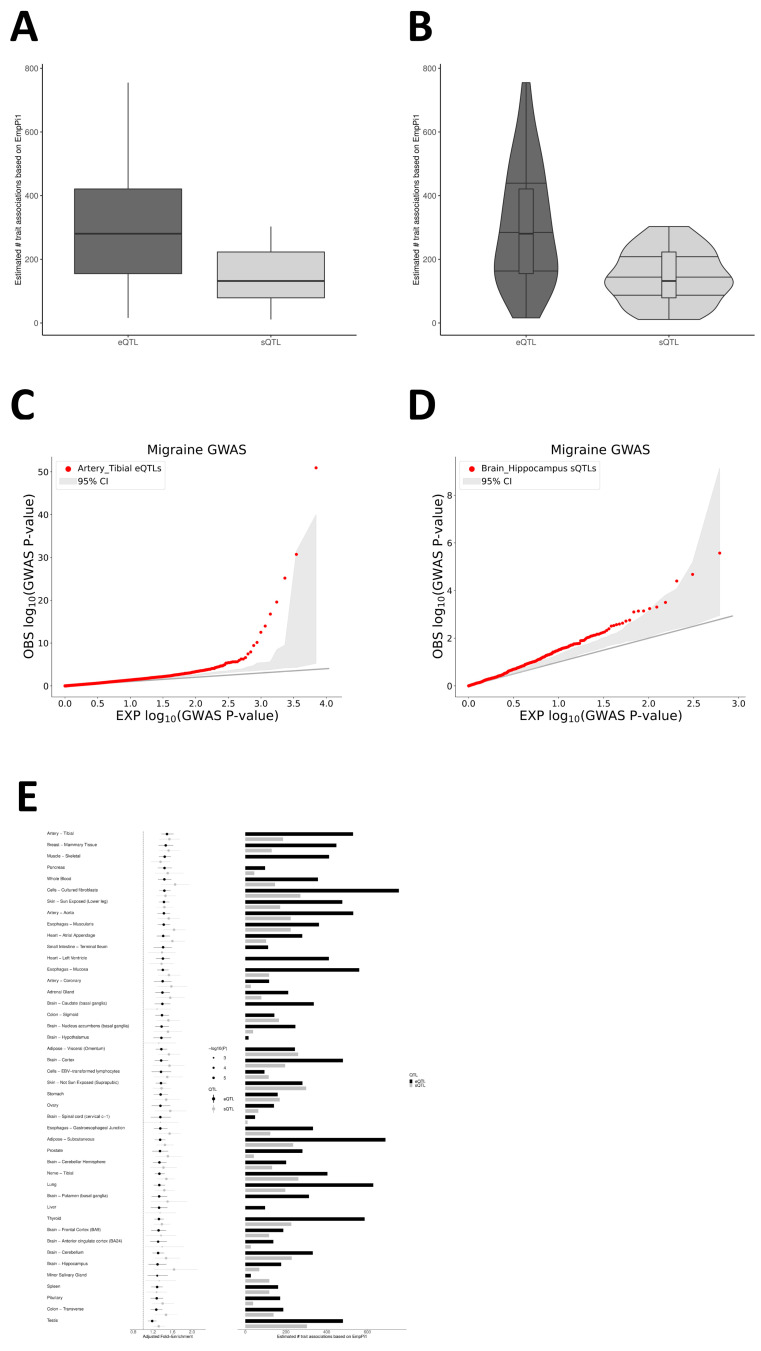
Tissue-level enrichment of migraine GWAS signals in regulatory QTLs. (**A**) Box plot showing the QTLEnrich-estimated number of migraine-associated regulatory variants per tissue across 49 GTEx v8 tissues, separated in eQTLs (dark gray) and sQTLs (light gray). The y-axis indicates the number of putative migraine-associated QTLs based on empirical π_1_ estimates. (**B**) Violin plots displaying the distribution of the QTLEnrich estimates for eQTLs (dark gray) and sQTLs (light gray) across the 49 tissues. (**C**) Quantile–quantile (QQ) plot for Artery—Tibial eQTLs. Red points represent observed −log_10_ (migraine GWAS *p*-values) at Tibial–Artery eQTL tag SNPs; the gray band indicates the matched null expectation. (**D**) QQ plot for Brain—Hippocampus sQTLs. Red points represent observed −log_10_(migraine GWAS *p*-values) at hippocampal sQTL tag SNPs; the gray band indicates the matched null expectation. (**E**) Tissue-wise summary of QTLEnrich results. Left: adjusted fold enrichment of migraine GWAS signals at eQTLs (dark gray) and sQTLs (light gray) with 95% confidence intervals. Right: QTLEnrich-estimated number of putative migraine-associated regulatory variants (empirical π_1_-based counts) for eQTLs (dark gray) and sQTLs (light gray).

To evaluate potential organ associations of eQTLs and sQTLs with migraine, we used the QTLEnrich method and adjusted for the significance and reliability of e/sQTL enrichment analysis results in QTLEnrich (Figure 1A–D). We examined 49 tissues from GTEx v8 to assess whether eQTLs and sQTLs showed non-random accumulation near migraine GWAS association signals (GWAS *p* < 0.05). Results indicate that both eQTLs and sQTLs exhibit significant enrichment in multiple peripheral vascular/circulatory-related tissues and central nervous system brain regions (Figure 1E; Supplementary Tables S1 and S2). At the sQTL level, enrichment is primarily concentrated in blood, vascular, and myocardium-related tissues, as well as brain regions closely related to pain modulation, stress response, and emotion evaluation, such as the hippocampus and other limbic/emotional regulatory structures, suggesting that splicing regulation in these tissues carries concentrated migraine genetic signals. In contrast, at the eQTL level, the most significant enrichments are distributed in peripheral arterial and cardiovascular-related tissues (e.g., tibial artery and aorta), accumulating substantial expression regulatory variants associated with migraine in these vascular smooth muscle tissues. These findings suggest that migraine-related genetic risks are not confined solely to the central nervous system or peripheral circulatory system but are simultaneously deposited in (i) splicing-level regulation in emotion–pain regulatory brain regions and (ii) expression-level regulation in peripheral vascular and cardiovascular tissues (Figure 1E; Supplementary Tables S1 and S2).

### 2.2. MAGMA Enrichment Analysis and Spatial Mapping Reveal Specific Histological Basis for Migraine

**Figure 2:**
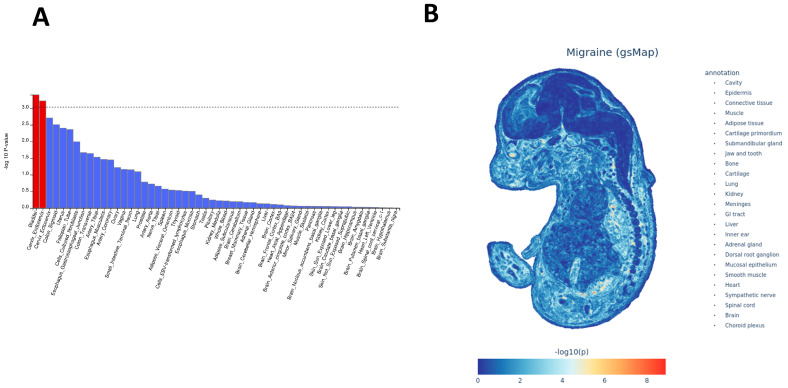
Tissue-level enrichment (MAGMA) and embryonic spatial mapping (gsMap) of migraine signals. (**A**) Bar plot of MAGMA tissue-specific expression enrichment across GTEx v8 tissues. Bars represent −log_10_(*p*) after multiple-testing correction. Red bars indicate tissues with significant enrichment that exceed the corrected significance threshold, while blue bars indicate non-significant tissues. The horizontal dashed line denotes the corrected significance threshold. Tissues are ranked by enrichment. (**B**) gsMap projection of migraine-associated genes onto the E16.5 whole-embryo spatial transcriptome. Color scale represents −log_10_(*p*), with the list of mapped anatomical regions shown on the right.

To expand the organ specificity suggested by QTLEnrich at the tissue level, we performed MAGMA tissue-specific expression enrichment using GTEx v8. Only Bladder and Cervix_Endocervix surpass the multiple-testing threshold (Figure 2A; Supplementary Table S3). Both tissues combine mucosal barrier and smooth muscle layers and are densely autonomic–sensory-innervated; the remaining top-ranked but sub-threshold tissues include gastrointestinal (GI) mucosa/Esophagus_Muscularis, peripheral arteries/vascular wall, and peripherally innervated structures. In contrast, most brain regions show trends but overall rank lower, suggesting that migraine gene-level enrichment is not limited to the central nervous system but preferentially accumulates in peripheral organs with mucosal–smooth muscle–vascular tone features. To resolve spatial localization in the developing organism, we projected migraine GWAS genes onto the E16.5 whole-embryo spatial transcriptomic atlas using gsMap. Signals are not uniform but appear as focal areas of higher concordance between trait signals and embryonic expression patterns (Figure 2B; Supplementary Table S4). These areas mainly fall into two recurrent patterns: (i) regions compatible with craniofacial and meningeal–trigeminal interfaces—such as jaw/tooth primordia, submandibular gland, nearby cartilage primordia, and meninges/choroid plexus—which are in line with known peripheral nociceptive entry points, and (ii) regions corresponding to visceral smooth muscle– and mucosa-like structures—including kidney, lung, gastrointestinal epithelium or mucosa, connective/cartilage primordia, heart, and smooth muscle-like layers—suggesting links to luminal-tone regulation and barrier biology. We also observe signals in structures annotated as dorsal root ganglion and sympathetic chain (i.e., peripheral sensory and autonomic components). Taken together, the gsMap projection indicates co-enrichment in trigeminal–meningeal and visceral smooth muscle–mucosal nodes rather than a cortex-restricted pattern, and this aligns with the peripheral-organ prioritization seen in MAGMA and with the vascular eQTL/selected brain sQTL signals observed with QTLEnrich.

### 2.3. Gene Function Enrichment Analysis Reveals Prioritized Tissue-Specific Biological Processes

To determine whether the tissue- and organ-level prioritization above is reflected at the pathway level, we performed GeneEnrich analyses on the GTEx v8 tissues that (i) show QTL-level enrichment for migraine (QTLEnrich) and/or (ii) are highlighted by MAGMA and gsMap, including arterial/heart tissues (Artery—Aorta, Artery—Tibial, Heart—Atrial Appendage, and Heart—Left Ventricle), Nerve—Tibial, several brain regions (Cortex, Anterior Cingulate Cortex BA24, Hypothalamus, and Hippocampus), and Whole Blood. For each tissue we used all detectable eQTL or sQTL genes of that tissue as the background (MHC excluded) and assessed significance by permutation-based empirical *p*-values (Supplementary Table S5).

**Figure 3:**
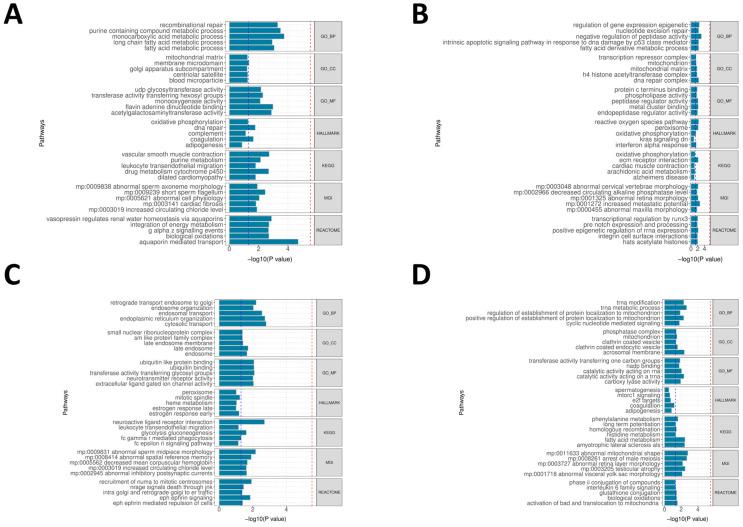
GeneEnrich functional enrichments for migraine across selected tissues. (**A**) Bar plot for Heart–Atrial Appendage (eQTL). Representative gene sets from databases (GO_BP/GO_CC/GO_MF, KEGG, Reactome, Hallmark, and MGI) are ranked by −log10(empirical *p*-value) on the x-axis. Dashed lines: blue at −log_10_(0.05) = 1.301; red, stringent cutoff at −log_10_(*p*) = 5.532. (**B**) Bar plot for Artery—Tibial (sQTL), formatted as in panel (**A**). (**C**) Bar plot for Nerve—Tibial (eQTL), formatted as in panel (**A**). (**D**) Bar plot for Brain—Hypothalamus (eQTL), formatted as in panel (**A**). Background sets include all detectable eQTL or sQTL genes per tissue, excluding MHC.

At the vascular–myocardial level, Heart—Atrial Appendage (eQTL) shows the most consistent signals, with enrichment of aquaporin-mediated transport, multiple lipid/monocarboxylate/purine metabolic processes, and KEGG vascular smooth muscle contraction at empirical *p* < 0.05 (Figure 3A). Artery—Aorta (eQTL) shows glycosylation/lysosomal-related pathways in GeneEnrich, consistent with the arterial signals highlighted by QTLEnrich, and Artery—Tibial (sQTL) shows a mixed pattern of peptidase/epigenetic-complex terms together with extracellular matrix and oxidative-phosphorylation pathways (Figure 3B), indicating that the arterial wall is one of the main sites where migraine-associated variants map onto coherent biological processes. In the peripheral sensory proxy, Nerve—Tibial (eQTL) is enriched for endosomal/endoplasmic reticulum transport and for KEGG neuroactive ligand–receptor interaction, accompanied by ligand-gated ion-channel activities (Figure 3C), which is compatible with the sensory–neuronal component already suggested by gsMap. Among brain tissues, the signals are overall weaker and more heterogeneous; BA24 (sQTL) mainly yields chromatin- and DNA-repair–related terms, whereas Hypothalamus (eQTL) is dominated by mitochondrial/tRNA processing and cell-cycle-related gene sets rather than classic neuroendocrine pathways (Figure 3D). Whole Blood (sQTL) additionally shows myeloid/leukocyte activation and interferon-related pathways at empirical *p* < 0.05. Taken together, these gene set results confirm that the migraine-relevant QTLs identified at the genome-wide and tissue levels converge most clearly in peripheral arterial/heart tissues and in the peripheral sensory compartment, while brain regions contribute smaller, pathway-specific signals (Figure 3A–D; Supplementary Table S5).

### 2.4. Single-Cell Transcriptomic Profiling and Cell-Type-Specific Enrichment in Migraine

**Figure 4:**
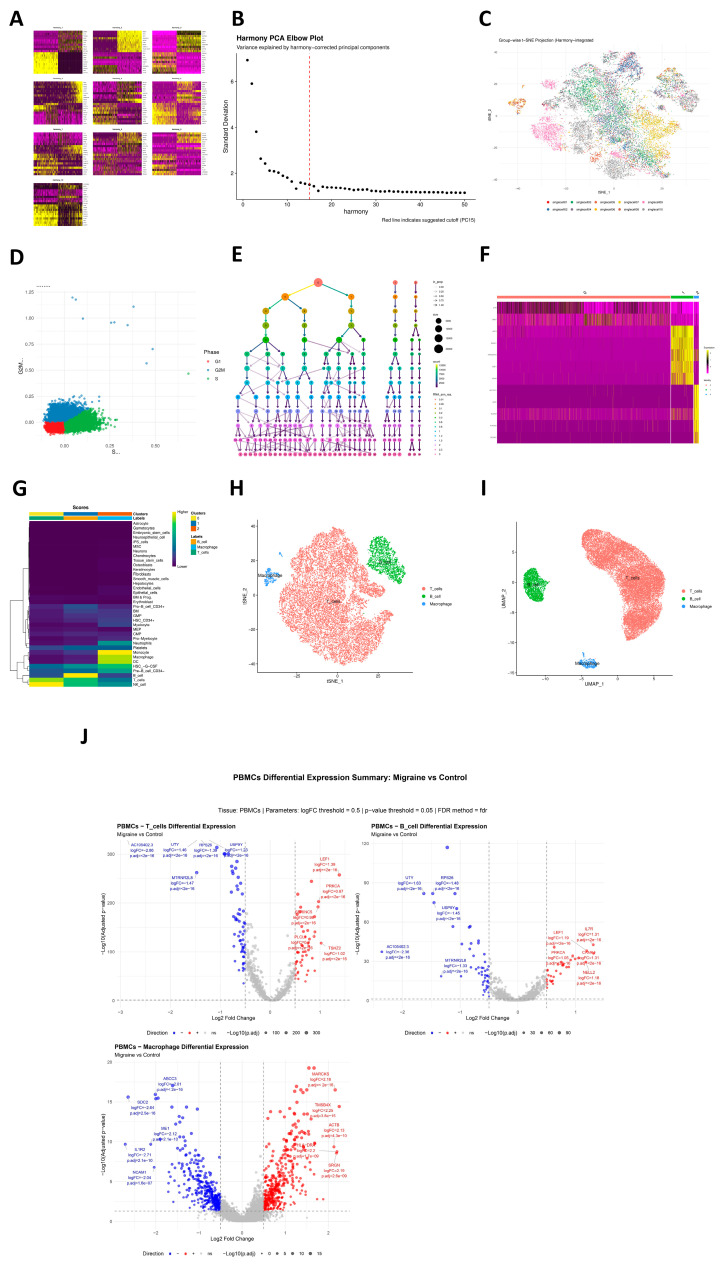
Integrated single-cell atlas, clustering, and cell-type annotation of the migraine dataset. (**A**) Principal component heatmap displaying weighted loadings of highly variable features across leading PCs. (**B**) Harmony PCA elbow plot: x-axis, Harmony-corrected PCs; y-axis, standard deviation; red dashed line marks suggested PC cutoff. (**C**) Harmony-integrated t-SNE embedding colored by sample/batch. (**D**) Cell-cycle scoring scatter plot (S.Score vs. G2M.Score) colored by phase (G1/S/G2M). (**E**) Clustering tree over multi-resolution community detection (0.01–3.0) on Harmony-based neighbor graph; resolution of 0.01 selected. (**F**) Cluster-specific marker gene heatmap (rows: markers; columns: cells ordered by cluster). (**G**) SingleR reference-based annotation score heatmap showing similarity to reference immune cell types. (**H**) t-SNE colored by final annotations (T cells in red, B cells in green, and macrophages in blue). (**I**) UMAP colored by final annotations (T cells in red, B cells in green, and macrophages in blue). (**J**) PBMC differential expression summary for migraine cases vs. controls. Volcano plots for T cells, B cells, and macrophages: x-axis, log_2_(fold change); y-axis, −log_10_(adjusted *p*-value); red points indicate up-regulated genes, and blue ones indicate down-regulated genes (logFC threshold 0.5, FDR < 0.05). All analyses were performed on high-quality cells with Harmony batch correction.

In the single-cell transcriptomic atlas annotation phase, based on Harmony-corrected low-dimensional embedding space, we performed systematic unsupervised clustering and cell-type identification on high-quality single-cell data. First, principal component heatmaps (Figure 4A) and elbow plots (Figure 4B) evaluated data dimensionality reduction quality, and t-SNE visualized Harmony-integrated cell distributions in multiple dimensions (Figure 4C), showing effective elimination of batch effects and clear biological structures in low-dimensional space. Further, S-phase and G2M-phase scoring quantified cell-cycle distribution (Figure 4D), revealing significant heterogeneity in cell-cycle phases across subpopulations, with some subsets showing evident cycle activity. On this basis, we built cell neighborhood graphs using corrected Harmony dimensionality reduction results and applied a multi-resolution clustering strategy (13 gradients from 0.01 to 3.0) for community identification. Clustering tree evaluation of stability across resolutions determined the optimal resolution of 0.01 for migraine (Figure 4E). Marker genes significantly highly expressed in each cell cluster were screened, and heatmaps were generated based on these genes (Figure 4F). Finally, the SingleR algorithm with reference datasets performed automated cell-type annotation (Figure 4G), successfully annotating three cell clusters as major immune cell types: T cells, B cells, and macrophages (Figure 4H,I). On this basis, we compared expression profiles of migraine cases versus controls within annotated T cells, B cells, and macrophages, detecting up- and down-regulated genes reaching pre-set thresholds in each cell type, indicating transcriptional-level differences within cell types in the case group despite overall consistent cell composition (Figure 4J). To examine whether migraine genetic risk preferentially localizes to peripheral immune cell types, we applied ECLIPSER to PBMC-derived populations. No cell type reaches significance after tissue-wide BH adjustment (Supplementary Table S6). At the point-estimate level, macrophages show a higher fold enrichment (1.73; 95% CI: 0.07–7.59; enrichment *p* = 0.326; BH-adjusted *p* = 0.977), whereas B cells and T cells are close to unity and slightly below one (B cells: 0.91; 95% CI: 0.59–1.00; *p* = 0.997; BH-adjusted *p* = 0.997; T cells: 0.91; 95% CI: 0.59–1.00; *p* = 0.997; BH-adjusted *p* = 0.997), indicating no clear PBMC cell-type-specific aggregation under the present data and settings (Supplementary Table S6). Using the CELLECT framework, we first applied S-LDSC to test cell-type-specific heritability enrichment (Supplementary Table S7). In the mousebrain panel, multiple endothelial subtypes reach nominal significance (ENTG3/ENTG2/ENTG4/ENTG1 and ENTG5/ENT7/ENTG6/ENTG7; strongest for ENTG3, *p* = 0.001), accompanied by vascular smooth muscle (VSMCA, *p* = 0.003), an inhibitory interneuron subset (TEINH18, *p* = 0.032), and a capillary/endothelial tag (VECA, *p* = 0.037). In the cross-tissue Tabula Muris panel, the top signals likewise converge on vascular wall constituents—heart smooth muscle cells (*p* = 1.47 × 10^−4^), brain pericytes (non-myeloid) (*p* = 2.25 × 10^−4^), heart myofibroblasts (*p* = 0.0018), and heart fibroblasts (*p* = 0.019). Notably, a bladder epithelial component (Bladder_bladder_cell, *p* = 0.015) is also observed, echoing our tissue-level MAGMA finding where the bladder epithelium survives multiple-testing correction (Figure 2A; Supplementary Table S3), thereby reinforcing the proposed “mucosa–smooth muscle–vascular tone” axis. Follow-up CELLECT–MAGMA analyses (Supplementary Table S8) broadly recapitulate endothelial/pericyte and smooth muscle patterns and show concordant nominal associations for inhibitory neuronal sets.

### 2.5. Integration of Multi-Dimensional Evidence for Cell Prioritization

To systematically prioritize key cell types most likely involved in migraine pathogenesis, we integrated three complementary computational biology evidence sources: (1) single-cell data annotation atlas analysis to identify active cell types in disease-related brain regions; (2) ECLIPSER analysis, evaluating GWAS signal enrichment in cell-type-specific regulatory elements based on functional genomics annotations; (3) CELLECT analysis, using S-LDSC and MAGMA to assess contributions of cell-type-specific expression genes to disease heritability. After availability adjustment, the vascular wall lineage achieves the most consistent support: CELLECT (across mousebrain and Tabula Muris) yields nominal associations for multiple endothelial subtypes (ENTG1–ENTG7) and vascular smooth muscle (VSMCA), with pericytes (non-myeloid) and heart smooth muscle/myofibroblast/fibroblast ranking highly (Supplementary Tables S7 and S8), all scoring a priority index of 1.00. An inhibitory interneuron subset (TEINH18) also reaches nominal significance in CELLECT (index of 1.00), supporting a central inhibitory circuit. In contrast, ECLIPSER on PBMC does not retain BH-significant enrichments; the atlas confirms T/B cells and macrophages as major populations, and macrophages show only directional evidence (fold enrichment 1.51; *p* = 0.363; BH ≈ 0.997), leading to an index of 0.50 for PBMC immune cells (atlas = 1, ECLIPSER = 0). Additionally, CELLECT S-LDSC indicates a bladder epithelial component at nominal significance, echoing our tissue-level MAGMA signal (Figure 2A; Supplementary Table S3). Collectively, the integrated scoring points to a recurrent involvement of vascular wall-related lineages and to additional, smaller signals in inhibitory neuronal and bladder epithelial cell types. These findings support a working model in which migraine risk variants may preferentially act through (i) peripheral vascular smooth muscle–mucosa-like tissues and (ii) selected central or sensory–autonomic cell populations, rather than a purely brain-restricted mechanism (Table 1).

### 2.6. Weighted Gene Co-Expression Network Analysis in PBMC T Cells

**Figure 5:**
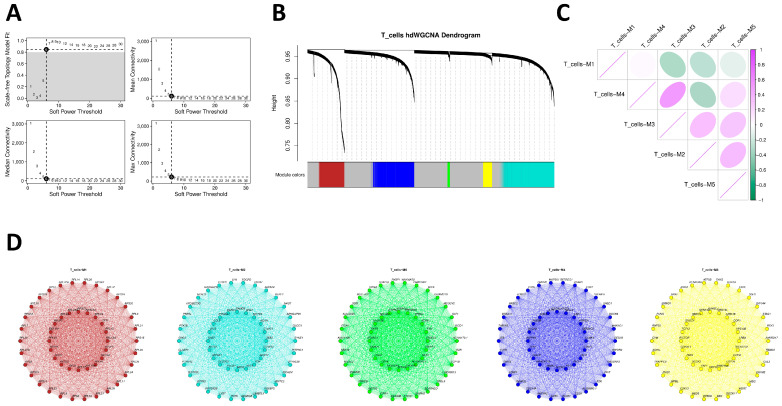
hdWGCNA of transcriptional programs in T cells. (**A**) Soft-threshold selection plots: top, scale-free topology model fit vs. soft power; bottom, mean connectivity vs. soft power; β = 6 selected. (**B**) Gene dendrogram with dynamic tree cut and module colors identifying M1–M5. (**C**) Module–eigengene (ME) correlation matrix. (**D**) Composite network views for modules M1–M5: nodes represent genes, and edges represent co-expression links (thresholded for clarity); representative hub genes are labeled. Color gradients indicate module-specific expression levels.

To resolve transcriptional regulatory structures within T cells in the peripheral immune compartment associated with migraine, we implemented hdWGCNA on PBMC T cells. After reducing sparsity with k = 25 metacells, soft-threshold scanning shows a robust scale-free fit plateau at β ≈ 6 while balancing connectivity, so β = 6 was selected for network construction (Figure 5A). Dynamic pruning hierarchical clustering identifies five co-expression modules (M1–M5) (Figure 5B), and module eigengene (ME) correlation heatmaps present clear inter-module association structures (Figure 5C). Functional interpretation shows M1 dominated by RPL/RPS/EEF1A1 and other ribosome/translation-related genes, presenting a housekeeping pattern; M2 centered on GNLY, NKG7, KLRD1, CCL5, TOX, NFATC2, and other typical effector/cytotoxic-related genes, also including PTK2B, VAV3, and others related to post-activation signaling or membrane/cytoskeleton dynamics, indicating that this module reflects not only cytotoxic/NK-like programs but also signaling components required for activated T cells; M3 enriched in DOCK2, RICTOR, GSK3B, BRAF, ARID1B, and other upstream signaling nodes and chromatin/transcriptional regulators; M4 enriched in CCR7, IL7R, LEF1, BACH2, FOXO1, and FOXP1, corresponding to naïve/central memory and migration/differentiation regulatory programs; M5 encompassing STAT5B, RASGRP1, PRKCB, TNFAIP3, ITGA4, CD84, and other T-cell receptor (TCR) signaling, negative feedback, and adhesion migration components. These reflect the multi-level transcriptional regulatory architecture of PBMC T cells in the peripheral immune environment (Figure 5D).

### 2.7. Co-Localization Analysis of Migraine GWAS Signals with e/sQTLs

**Figure 6:**
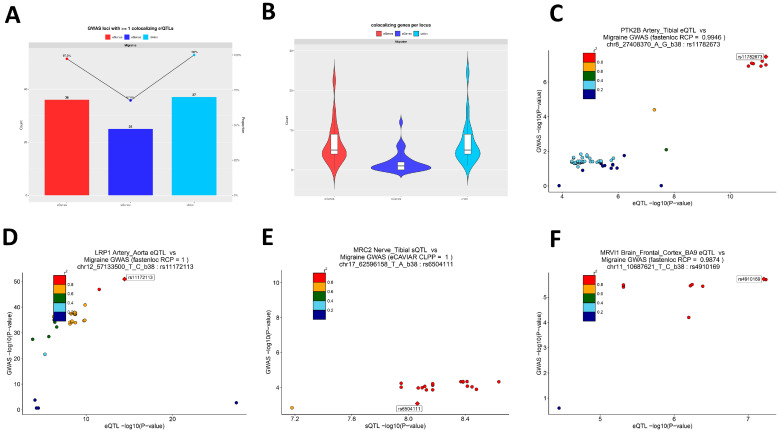
Co-localization of migraine GWAS loci with regulatory QTLs. (**A**) Bar-and-line plot showing the number (left y-axis) and proportion (right y-axis) of 37 FUMA-defined migraine GWAS lead loci with significant co-localization to eQTLs (red), sQTLs (blue), or either (cyan) across 49 GTEx v8 tissues. (**B**) Violin plots of co-localized gene counts per GWAS locus for eQTL-derived genes (red), sQTL-derived genes (blue), and union (cyan). (**C**) Scatter plot comparing migraine GWAS −log10(*p*-value) and GTEx Artery—Tibial eQTL −log10(*p*-value) at the PTK2B locus (lead variant rs11782673); point colors denote LD (r^2^) with the lead eQTL; fastENLOC RCP = 0.9946. (**D**) Scatter plot for LRP1 Artery—Aorta eQTL vs. migraine GWAS (lead rs11172113); fastENLOC RCP = 1. (**E**) Scatter plot for MRC2 Nerve—Tibial sQTL vs. migraine GWAS; best variant rs6504111; eCAVIAR CLPP = 1. (**F**) Scatter plot for MRVI1 Brain—Frontal Cortex (BA9) eQTL vs. migraine GWAS; best variant rs4910169; fastENLOC RCP = 0.9874.

To map migraine GWAS signals to actionable transcriptional regulatory levels, we first used FUMA on the primary analysis GWAS results for risk locus identification, obtaining 37 lead loci for subsequent functional validation (Supplementary Table S9). We then co-localized these 37 loci with eQTL/sQTL data from 49 GTEx v8 tissues using eCAVIAR (threshold CLPP > 0.01) and fastENLOC (threshold RCP > 0.10) across tissues. Results show that among the 37 FUMA-defined migraine GWAS loci in Figure 6A, 36 loci have at least one significant co-localization with eQTL in one tissue and 25 loci with sQTL, and all 37 loci have at least one co-localization evidence at e- or sQTL level, indicating that most migraine genetic signals can be linked to actual expression or splicing regulatory variants (Figure 6A). At the gene level, eCAVIAR co-localizes 169 unique genes (eGenes = 155; sGenes = 45) and fastENLOC 214 unique genes (eGenes = 199; sGenes = 58), with 129 intersection genes and 254 union genes (Supplementary Tables S10–S12). Figure 6B presents the distribution of co-localizable gene counts per GWAS locus, divided into eQTL-sourced genes, sQTL-sourced genes, and union after merging methods, with union median of 5 (interquartile range of 4–9), reflecting that most loci correspond to more than one candidate effector gene; when integrating eCAVIAR and fastENLOC results at the table level for the same locus, cross-validation converges candidate lists to fewer, recurring genes (Supplementary Table S12). From tissue and QTL-type distributions, the most consistent co-localization signals concentrate in vascular and myocardial-related tissues (Artery—Aorta, Artery—Tibial, Heart—Atrial Appendage, and Heart—Left Ventricle), aligning with the “peripheral vascular wall regulatory axis” inferred from prior enrichment and functional annotations; additionally, strong splicing-level co-localization is detected in the peripheral nerve tissue Nerve—Tibial, indicating that RNA processing in peripheral sensory–pain-related tissues may be a landing point for migraine genetic effects; at the central level, robust eQTL co-localization is observed in multiple cerebral cortical regions, supporting that migraine also transmits genetic risk through central pain–emotion–autonomic regulatory pathways. To demonstrate the specific forms of these three biological axes, we selected 4 loci with strong statistical evidence consistent with prior results from the 37 loci for regional co-localization plots (Figure 6C–F). First, rs11782673 (PTK2B) shows high consistency between GTEx Artery—Tibial eQTL and migraine GWAS signals, with fastENLOC-estimated regional co-localization probability RCP = 0.9946 (Figure 6C); similar co-localization strength in Artery—Aorta (RCP = 0.9853); and the same allelic expression regulatory direction in Heart—Atrial Appendage, Heart—Left Ventricle, and other cardiovascular tissues. This indicates that this locus is a typical multi-tissue consistent vascular expression regulatory risk signal. Further, eCAVIAR shows co-localization for the same GWAS SNP with PTK2B sQTL in multiple brain regions (Brain Cortex, Frontal Cortex BA9, Hippocampus, and Nucleus accumbens), suggesting that the locus may transmit genetic effects not only at peripheral vascular–myocardial levels but also via splicing regulation in central pain- or emotion-related regions. Second, rs11172113 (LRP1) in Artery—Aorta eQTL achieves extremely high regional co-localization probability according to fastENLOC (RCP = 1), consistent with observations in other arterial/myocardial tissues, classifying this locus into the vascular–myocardial regulatory module (Figure 6D). At the peripheral nerve level, in the GWAS locus indexed by rs11657101, the best co-localized variant for Nerve—Tibial MRC2 sQTL is rs6504111, with eCAVIAR CLPP = 1.0, indicating that the locus’ genetic effect can be realized through splicing regulation in peripheral nerves, supplementing functional evidence at the splicing level (Figure 6E). Finally, in central nervous tissues, the locus at rs4910165 shows the best co-localized variant, rs4910169, for Brain—Frontal Cortex (BA9) MRVI1 eQTL, with fastENLOC RCP = 0.9874, suggesting that this migraine genetic signal lands on cortical-level expression regulation (Figure 6F). Results suggest that both expression and splicing as tissue-specific regulation mechanisms are important molecular mechanisms mediating GWAS signals.

### 2.8. Cis-Regulatory Analysis Using Open4Gene Hurdle Regression

Using the Open4Gene hurdle regression framework, we systematically screened cis-peak–gene regulatory relationships in the CD4 central memory T (TCM) cell population using the healthy donor multi-omics atlas. This analysis aimed to define the baseline cis-regulatory potential of migraine risk loci in a physiological immune context. First, results underwent cell count QC (gene expression cells ≥ 10 and peak openness cells ≥ 10), followed by Benjamini–Hochberg multiple correction on the model’s count component *p*-values within the same cell type. Results identify 1763 significant cis-peak–gene regulatory pairs in CD4 TCM (false discovery rate [FDR] < 0.01), further requiring high expression support for the gene in the cell population (expression cells ≥ 50), and peak openness cells ≥ 20 yields 751 high-confidence regulatory pairs (Supplementary Table S13). This set includes multiple genes consistent with T-cell transcriptional programs, such as C2CD2L, TMEM101, TMEM242, LINC00324, and PITPNM1, with peak openness and gene expression levels showing significant positive associations in CD4 TCM (count component FDR < 0.01), suggesting that these genes have accessible cis-regulatory backgrounds in CD4 memory-like T cells with circulatory/re-activation features.

### 2.9. Spatial Distribution of Migraine-Related Genes Using gsMap

**Figure 7:**
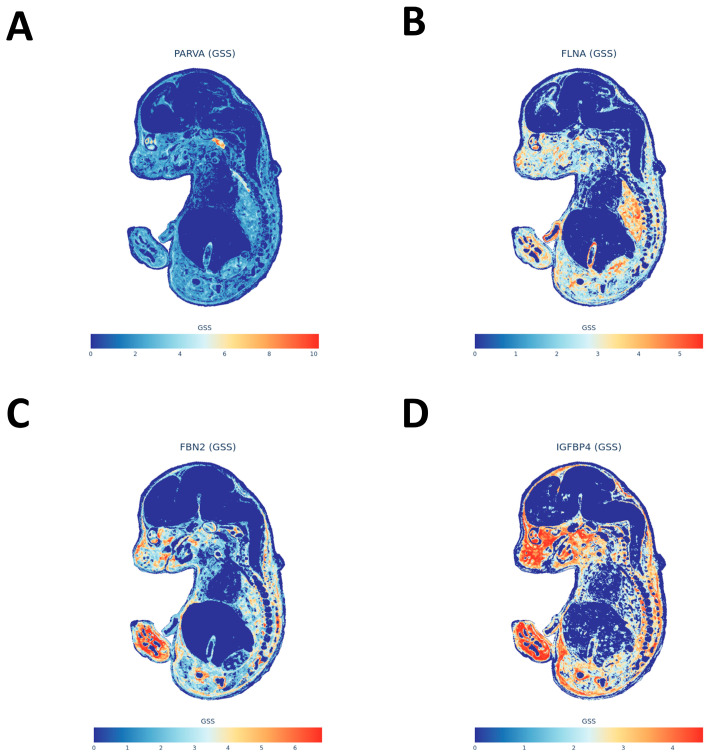
gsMap-derived spatial distributions of representative migraine-associated genes in the E16.5 embryo. (**A**) Spatial map of PARVA (GSS). Color scale from blue (low) to red (high) indicates gene-specificity score (GSS). (**B**) Spatial map of FLNA (GSS), formatted as in panel (**A**). (**C**) Spatial map of FBN2 (GSS), formatted as in panel (**A**). (**D**) Spatial map of IGFBP4 (GSS), formatted as in panel (**A**).

After ranking gene-level gsMap diagnosis results by spatial specificity scores, high-scoring genes are not uniformly distributed but mainly concentrated in two embryonic structures: one corresponding to smooth muscle, vascular wall, and interstitial/connective tissues related to peripheral lumens and the other to sympathetic or peripheral nerve chains and craniofacial cartilage precursors related to meningeal–nerve transitions (Supplementary Table S14). To visually display these two spatial signals, we selected four representative genes from the top-ranked ones for spatial expression mapping: PARVA forms focal enrichments in embryonic sympathetic nerve chains and related ganglia, showing spatial landing points for autonomic–sensory pathways (Figure 7A); FLNA presents continuous distribution in smooth muscle and vascular-like tissues, consistent with peripheral vascular smooth muscle regulation (Figure 7B); FBN2 appears in focal aggregations in craniofacial cartilage precursors and surrounding mesenchyme, suggesting that craniofacial developmental structures also carry partial genetic signals (Figure 7C); IGFBP4 localizes to jaw/facial/tooth primordia-adjacent areas, supplementing spatial evidence for meningeal/craniofacial interfaces (Figure 7D). Overall, spatial distributions of these high-scoring genes are compatible with the “peripheral mucosa–smooth muscle–vascular tone axis” and “peripheral sensory/sympathetic–meningeal node” inferred from functional enrichment, co-localization, and immune cell resolution, supported in developmental spatial atlas form (Supplementary Table S14; Figure 7A–D).

### 2.10. Expression Annotation and Exon-Level Analysis of Preferred Genes

**Figure 8:**
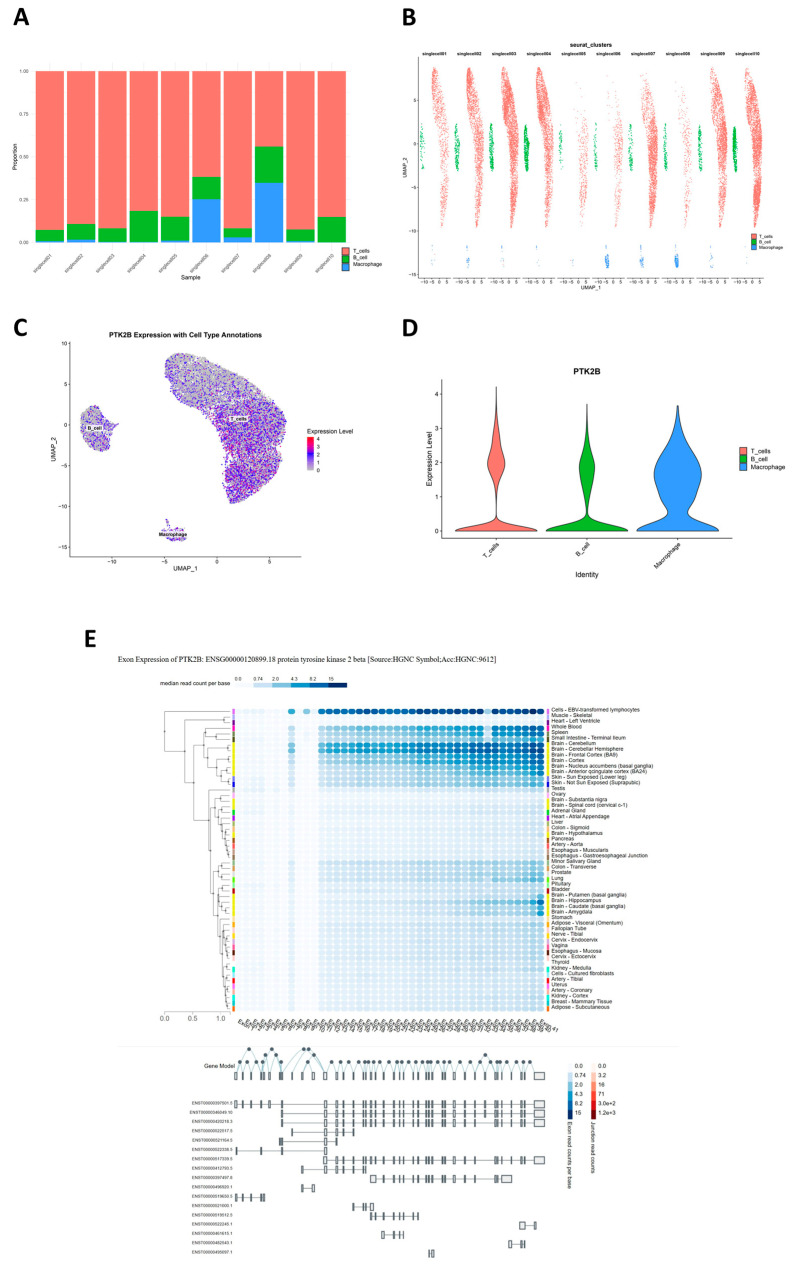
Single-cell and exon-level expression of PTK2B. (**A**) Stacked bar plot showing cell composition proportions across 10 samples (migraine cases: singlecell01–05; controls: singlecell06–10). Colors: T cells (red), B cells (green), and macrophages (blue). (**B**) Sample-wise UMAP panels showing the distribution of T cells (red), B cells (green), and macrophages (blue) for 10 PBMC samples. (**C**) UMAP plot of single-cell data showing PTK2B expression overlaid on T cells, B cells, and macrophages; color scale from purple (low) to yellow (high) indicates expression level. (**D**) Violin plot comparing PTK2B expression distribution among T cells (red), B cells (green), and macrophages (blue). (**E**) GTEx exon-level expression heatmap with PTK2B transcript structure. Rows: tissues; columns: exons; color scale indicates median read count per base (blue (low) to red (high)). Bottom: exon structure with junctions.

First, to assess consistency of single-cell data across subjects, we compared cell compositions in 10 samples. Stacked bar charts show all samples primarily composed of T cells, followed by macrophages, with B cells the lowest; migraine cases (singlecell01–05) and normal controls (singlecell06–10) are largely consistent in overall immune cell composition, with only mild macrophage proportion increases in individual migraine samples and no systematic cell group shifts due to disease grouping (Figure 8A). Grouped UMAP also shows three major immune cell populations clearly separated in each sample, with cells from different sources mixing well within clusters, indicating good batch effect control in integrated data for subsequent gene-level expression annotation (Figure 8B). From integrated cross-tissue co-localization results, PTK2B shows consistent eQTL and sQTL co-localization not only in peripheral tissues like arteries but also in multiple cerebral cortical regions and is assigned to activation-related modules in PBMC T-cell hdWGCNA; thus, we further performed single-cell-level expression annotation for PTK2B. Integrated single-cell UMAP expression projection shows PTK2B detectable in T cells, B cells, and macrophages, not limited to a single cell type; some T cells show higher expression, macrophages are mostly in the medium expression range, and B cells show overall lower expression (Figure 8C). Corresponding violin plots also show T cells with the highest expression upper limit, macrophages with wider moderate expression distribution, and B cells at low expression levels, supporting relatively more significant expression preference for PTK2B in adaptive immune cells (Figure 8D). In exon-level expression annotation, we used GTEx exon expression data for PTK2B transcript structure and tissue distribution analysis. Results show PTK2B exons with continuous and sufficient read coverage in immune-related cells and most central nervous system tissues, with Epstein–Barr virus (EBV)-transformed lymphocytes showing high read depths for nearly all exons, suggesting that this cell type expresses PTK2B mainly in near-full-length transcript form. Multiple brain regions (e.g., cerebellum, frontal cortex, nucleus accumbens, and caudate) also show medium to high exon signals; combined with transcript structures, some brain tissues may preferentially use mid-to-late exons, possibly reflecting tissue-specific splicing patterns of PTK2B in neural signaling and immune-related pathways (Figure 8E).

### 2.11. Identification and Conditional Analysis of Migraine Risk Loci

From the IHGC 2022 migraine summary statistics (excluding 23andMe), we identified 37 independent genome-wide significant loci (*p* < 5 × 10^−8^) using the FUMA pipeline, distributed across multiple autosomes (Supplementary Table S9). Chromosome 6 has the most concentrated signals, detecting six independent risk loci: rs10456100, rs11153082, rs34273564, rs6904682, rs7757975, and rs9349379; chromosomes 1 and 10 each detect four risk loci, indicating these chromosomes as important genetic enrichment regions. To further resolve whether independent secondary pathogenic variants exist within each GWAS locus, we implemented GCTA-COJO conditional analysis on segments containing the 37 lead SNPs, using a conditional *p*-value (pC) < 0.05 as the criterion for screening potential secondary signals. Results yield 2594 qualifying variants distributed across 34 loci, mainly in segments with the strongest original associations and more complex LD structures, such as loci represented by rs11153082 (chr6), rs72926788 (chr2), rs2274224 (chr10), and rs13078967 (chr3) (Supplementary Table S15). After tightening the criterion to pC < 5 × 10^−8^, only the chromosome 1 q32 segment led by rs2078371 retains a cluster of variants reaching genome-wide significance post-conditioning, suggesting this region harbors multiple functional variants and is one of the most complex genetic architecture risk genomic loci in this study (Supplementary Table S15). Overall, results show that the trait’s genetic basis consists, on one hand, of a few highly significant, chromosome-specific clustered main-effect loci and, on the other hand, of latent secondary independent signals in several key loci revealed only by conditional analysis, reflecting a multi-level and regionally varying genetic structure.

## 3. Discussion

Our study provides a comprehensive multi-scale perspective on how common genetic risk factors map onto the biology of migraine. Consistent with the view of migraine as a complex neurovascular and neuroinflammatory disorder involving neurons, glia, vasculature, and immune elements [16,17,18,19], we found that migraine-associated variants converge on pathways and tissues far beyond the brain alone. This aligns with growing evidence that migraine pathophysiology extends to systemic dysregulation, including immune activation and metabolic disturbances. A recent scoping review summarized consistent abnormalities in pro- and anti-inflammatory cytokines and other immune mediators in people with migraine, both during and outside attacks [18], while a multi-omics Mendelian randomization analysis proposed a “lactylation–immune regulatory axis” in which lactate-derived histone and protein lactylation modulates immune traits and migraine risk [20]. Epidemiologically, several immune-mediated diseases, such as rheumatoid arthritis, show an elevated subsequent risk of migraine [21]. At the same time, neuromodulatory peptides like CGRP and pituitary adenylate cyclase-activating polypeptide (PACAP) bridge vascular, neural, and immune signaling and are now key drug targets [22]. Notably, in a real-world cohort of patients with migraine and concomitant immune disorders, combined use of anti-CGRP monoclonal antibodies and disease-modifying immunomodulatory therapy was associated with better migraine outcomes in this real-world cohort compared with monotherapy [23]. Together, these observations support a neuroimmune–metabolic framework for migraine and underscore the need to integrate central and systemic perspectives in mechanistic studies and therapeutic strategies.

Genome-wide association studies have firmly established that migraine is highly polygenic. The largest migraine meta-analysis to date identified 123 risk loci and showed that migraine-associated variants are enriched in both vascular tissues and central nervous system cell types, reinforcing a neurovascular model of the disorder [14]. However, translating these loci into specific effector genes, tissues, and pathways requires functional context. Our work addresses this gap by integrating GWASs with tissue QTLs, single-cell transcriptomic and multi-omics data, chromatin accessibility, and spatial transcriptomics to construct a system-wide atlas of migraine genetic risk from organs to cell types and molecular mechanisms. This complements more focused efforts that emphasize particular biological layers. For example, a recent single-cell multi-omics framework highlighted immune cell subsets and transcriptional programs that may drive migraine and suggested repurposable immune-modulating therapeutics [24]. While that study concentrated on peripheral immune cells, our integrative strategy systematically surveyed diverse tissues and annotated cell types to build a broader map.

At the tissue level, we observed that migraine risk variants are non-randomly concentrated in eQTLs and sQTLs from peripheral arteries (e.g., tibial artery and aorta), heart, tibial nerve, selected brain regions, and whole blood. This pattern suggests that common migraine variants exert substantial effects on vascular and smooth muscle biology, as well as on peripheral sensory- and central pain-modulatory circuits. The finding that arterial and myocardial tissues show the most robust eQTL co-localization and pathway enrichment aligns with prior GWAS-based evidence of vascular involvement [14] and underscores the importance of the vascular wall as a site where migraine risk alleles manifest their effects. Our MAGMA further highlighted tissues that combine mucosal, smooth muscle, and autonomic-innervation features—such as the bladder and the cervix endocervix. We interpret these findings with caution, as the biological link between these organs and migraine is not fully established. Rather than implying direct organ pathology, these signals likely reflect shared underlying biological properties, such as smooth muscle contractility and autonomic regulation. Consistent with this view, sub-threshold but top-ranked tissues included esophageal muscularis and other gastrointestinal mucosal structures. Complementary spatial transcriptomic mapping using the MOSTA atlas likewise pointed to visceral smooth muscle, peripheral autonomic and sensory ganglia, and craniofacial/meningeal interfaces. We acknowledge that migraine typically manifests in adolescence or adulthood; thus, these embryonic spatial signals should be interpreted through the lens of “developmental priming.” We hypothesize that genetic risk variants expressed during the organogenesis of the trigeminovascular system and autonomic ganglia may compromise their structural integrity or wiring. This developmental “imprinting” could establish a latent neurovascular vulnerability that predisposes individuals to migraine later in life, consistent with the developmental origins of health and disease framework. These signals are notable because gastrointestinal and genitourinary symptoms are common in migraine, and irritable bowel syndrome (IBS) in particular shows bidirectional comorbidity with migraine, with odds ratios around two to two-and-a-half in both directions in the meta-analysis [25]. Such patterns are compatible with a shared gut–brain/autonomic dysregulation, in which mucosa–smooth muscle–vascular tone axes are co-affected in migraine and IBS [26,27,28]. In addition, we observed spatial enrichment in regions corresponding to cranial meninges, trigeminal nerve territories, and related craniofacial mesenchyme, consonant with the established trigeminovascular model in which meningeal vessels and their innervation are central generators of migraine pain [29]. Taken together, the tissue and spatial results support a view of migraine as a multisystem disorder engaging peripheral vascular and mucosal organs, autonomic and sensory nodes, and specific central structures, rather than a cortex-restricted brain disease.

The pleiotropic nature of migraine genetics was further evident at the pathway and cell-type levels. Gene set enrichment analyses in migraine-relevant tissues (arteries, heart, tibial nerve, selected brain regions, and whole blood) highlighted several recurring biological themes: (i) vascular smooth muscle contraction and cytoskeletal or extracellular matrix processes, implicating regulation of vessel tone and structural integrity; (ii) metabolic and mitochondrial pathways, including oxidative phosphorylation and monocarboxylate/lipid metabolism, consistent with a role for bioenergetic stress and metabolic by-products in modulating neural excitability and neuroinflammation; (iii) neuroactive ligand–receptor interaction and ion-channel activity, highlighting neurotransmitter, neuropeptide, and neuromodulator systems; and (iv) immune and inflammatory pathways, including leukocyte activation and interferon signaling, indicating that both innate and adaptive immune processes contribute to migraine susceptibility. These pathway-level findings resonate with current pathophysiological models in which vascular reactivity, CGRP/PACAP signaling, mitochondrial function, and inflammatory mediators all shape migraine attack thresholds and phenotypes [16,30,31]. In particular, the enrichment of immune pathways is in line with comprehensive evidence from clinical and experimental studies that migraine is associated with altered cytokine profiles, complement components, and immune cell function [18,32].

In terms of specific cell types, our heritability partitioning and cell-type enrichment analyses consistently prioritized vascular wall cell lineages. Using CELLECT, migraine-associated variants showed significant enrichment in multiple endothelial subtypes, vascular smooth muscle cells, pericytes, and related fibroblast/myofibroblast populations across independent reference panels, whereas brain cell-type signals were more modest and selective, with nominal enrichment in an inhibitory interneuron subset. This pattern supports the concept of a “neurovascular unit” in which vessel wall cells and local neural/glial elements jointly integrate genetic risk [33,34]. The inhibitory interneuron signal also aligns with evidence for cortical hyperexcitability and impaired inhibition, particularly in migraine with aura, and suggests that genetic variants influencing inhibitory circuits may modulate thresholds for spreading depolarization and sensory hypersensitivity [35,36].

By contrast, our analyses did not identify significant heritability enrichment in any specific peripheral blood immune cell type, and ECLIPSER did not detect BH-significant enrichment of GWAS signals in PBMC-derived regulatory annotations. This absence of strong enrichment in circulating leukocytes is consistent with a recent Mendelian randomization study that found no evidence for a causal effect of major autoimmune diseases on migraine risk or for substantial genetic correlation between them [37], despite the epidemiological co-occurrence of migraine with conditions such as rheumatoid arthritis [21]. Reflecting this, our integrated prioritization system assigned a lower priority index (0.5) to PBMC subsets compared with vascular lineages (1.0). However, the lack of global heritability enrichment does not preclude specific risk genes from functioning within immune cells. Nonetheless, hdWGCNA in T cells revealed co-expression modules enriched for cytokine signaling, cytotoxicity, and T-cell receptor signaling, within which some co-localized genes (such as PTK2B) reside. These modules may reflect genetically primed immune programs that are activated under specific stimuli in migraine, a possibility that warrants further functional work and dovetails with clinical data on the benefit of immune-targeted co-therapies in selected patients [22].

By integrating FUMA locus discovery with Bayesian co-localization (eCAVIAR, fastENLOC) across 49 GTEx tissues, we were able to nominate candidate effector genes for nearly all migraine GWAS loci. At 36 of 37 loci, we identified at least one co-localizing eQTL gene and at 25 loci at least one sQTL gene, with most loci harboring multiple plausible effectors. This supports a model in which common variants act largely through perturbations of gene expression or splicing in specific tissues. Importantly, many of the prioritized genes have functions that are highly compatible with migraine biology. As a representative example of this multi-omics convergence, PTK2B (encoding the non-receptor tyrosine kinase PYK2) showed strong co-localization with eQTLs in arterial and myocardial tissues as well as sQTLs in multiple cortical regions and was embedded within an activation/cytotoxicity module in T cells. PYK2 is a calcium-sensitive kinase involved in integrin, neurotransmitter, and immune signaling, offering a mechanistic link among vascular, neural, and immune compartments that is coherent with our multi-scale findings [38,39]. LRP1, which we identified through arterial eQTL co-localization at a genome-wide significant locus, encodes a multifunctional endocytic receptor that influences lipid trafficking, endothelial barrier function, and cell signaling [40]. Recent work has shown that the LRP1–SHP2 pathway modulates TRPV1 sensitivity in peripheral nociceptors and that amyloid β1–42 can alter this axis to change pain thresholds [41]. These data provide convergent support for LRP1 as a modulator of nociceptive processing at the neurovascular–neural interface, consistent with its prioritization in our vascular-focused analyses. Another example is MRVI1, which we found to co-localize with cortical eQTLs at a migraine risk locus. MRVI1 is involved in NO–cGMP–IP3 receptor signaling and regulates intracellular calcium release in smooth muscle cells and neurons, placing it in a pathway central to vasodilation and excitability [42,43,44]. Taken together, such genes illustrate how integrating GWAS with tissue QTLs and cell-level data can refine effector candidates from broad loci to specific molecules with testable roles in migraine pathophysiology.

Our findings also align with and extend recent integrative genomics work that has begun to propose druggable targets in migraine. A multi-omics Mendelian randomization study integrating GWAS, eQTL, and protein quantitative trait loci (pQTL) data highlighted GSTM4, a glutathione S-transferase gene involved in oxidative stress responses, as a promising therapeutic target [45]. Separately, machine learning-driven analysis of gene programs in migraine identified key transcription factors and regulatory modules enriched for synaptic and calcium-signaling pathways, with additional involvement of inflammatory processes [46]. Moreover, analysis of a familial migraine–epilepsy phenotype combined with GWAS information has implicated NCOR2, a nuclear corepressor that coordinates inflammatory and neuronal gene expression programs, as a candidate gene linking neuronal hyperexcitability and migraine susceptibility [47]. These independent studies converge with our observation that migraine genes cluster in networks governing ion channels, second-messenger cascades, synaptic function, and immune–metabolic regulation. Importantly, several clinically validated targets, such as CALCA/CALCB (CGRP ligands) and HTR1F (5-HT1F receptor), reside in migraine loci in the large GWAS [14]. The fact that unbiased genetic and functional datasets repeatedly point to targets already proven by pharmacology lends weight to newly prioritized genes like PTK2B, LRP1, MRVI1, and GSTM4, which may represent the next generation of mechanistic and therapeutic candidates.

Several limitations of our approach warrant consideration. First, most of the QTL and single-cell reference datasets we used were generated from non-migraine donors and represent baseline regulatory architecture rather than disease or attack states. While such resources are indispensable for mapping genetic effects, they cannot capture dynamic transcriptomic or epigenomic changes during migraine attacks, nor can they reflect the disease-driven remodeling of tissues. Future studies incorporating genotype-informed omics in migraine patients—ideally sampling key tissues such as trigeminal ganglia, meninges, and hypothalamus during relevant phases—will be crucial to adding disease-context specificity. Second, tissue coverage remains incomplete. Critical migraine-relevant structures, including meningeal arteries, trigeminal ganglia, specific brainstem nuclei, and hypothalamic subnuclei, are absent or sparsely represented in current eQTL and single-cell QTL catalogs. Our reliance on proxies (e.g., tibial nerve for peripheral nociceptors and major arteries for meningeal vessels) introduces uncertainty and may obscure highly localized effects. Expanding QTL and single-cell resources to these specialized tissues and cell types is therefore a priority for the field. Third, our single-cell analyses of immune cells were limited to PBMCs in a modest-sized case–control cohort. While we did not observe clear genetic enrichment in peripheral immune subsets, this does not exclude important roles for tissue-resident immune cells, nor does it capture non-genetic drivers of immune activation in migraine. Integrating larger, ethnically diverse cohorts with the multi-omics single-cell profiling of central and peripheral neuroimmune interfaces will help resolve these questions. Fourth, the gsMap-based projection of human migraine GWAS data onto a mouse embryonic spatial atlas is inherently indirect. While this approach effectively identifies the developmental origins of susceptible tissues, embryonic expression patterns may only partially recapitulate adult tissue architecture and disease-relevant states. Therefore, these spatial enrichments likely reflect the genetic establishment of structural susceptibility (e.g., vascular tone or neural connectivity) rather than the active pathophysiology of a migraine attack. We therefore interpret spatial hotspots as hypothesis-generating, pointing to candidate organ systems (e.g., craniofacial mesenchyme, sympathetic chain, and visceral smooth muscle) rather than as definitive localization. Finally, our analyses, like all polygenic studies, describe probabilistic shifts in pathway activity and tissue liability, not deterministic outcomes. Migraine risk alleles increase susceptibility but operate in concert with environmental exposures, hormonal factors, and epigenetic mechanisms. Experimental validation—through gene editing, pharmacologic perturbation, and in vivo models—is essential to confirming causal roles for the genes and pathways we have prioritized and to delineate their interactions.

## 4. Materials and Methods

### 4.1. Study Design and Ethics

**Figure 9:**
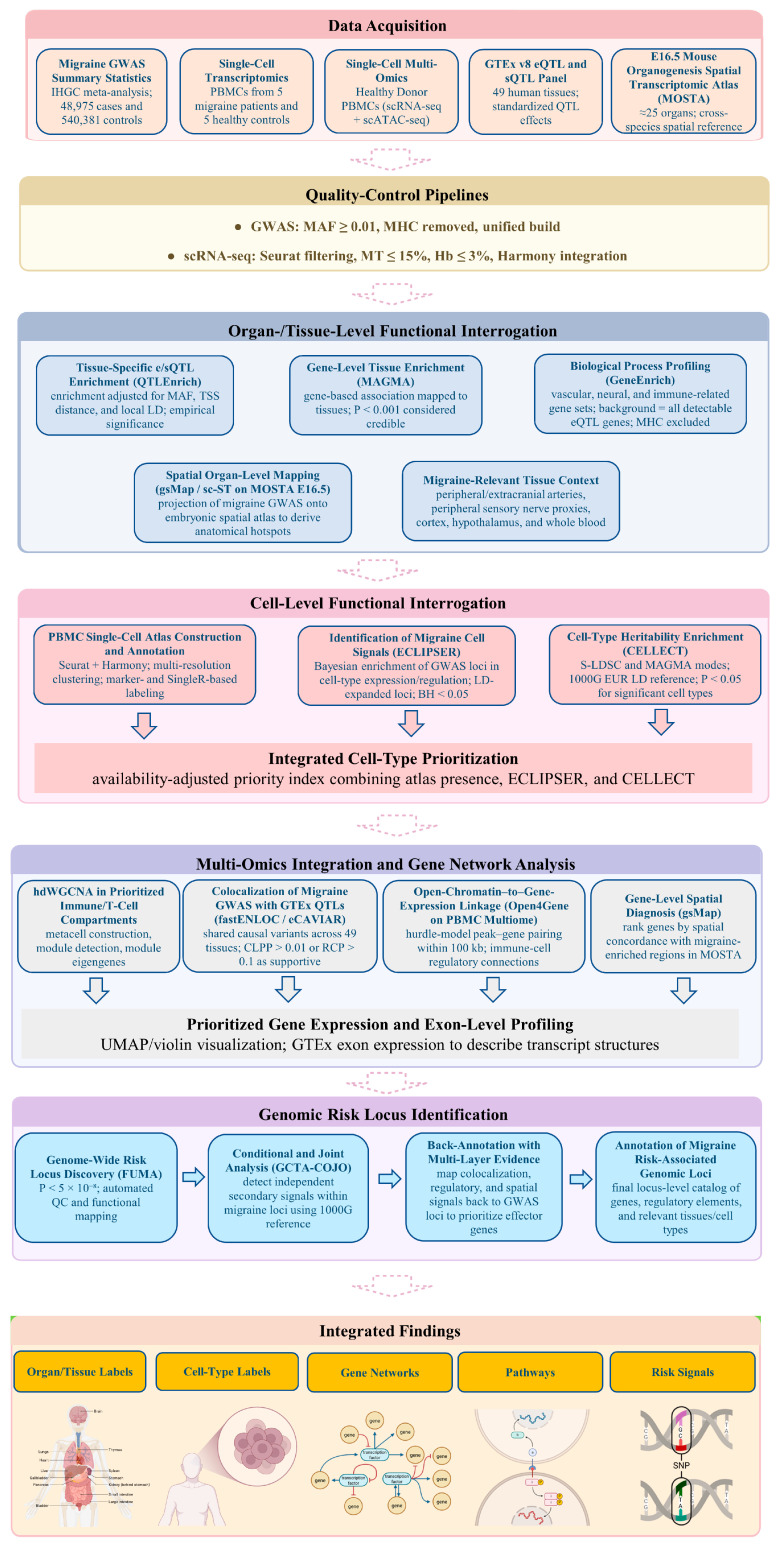
Study design and multi-layer framework for mapping migraine genetic risk from organs to cell types and genes.

All data included in this study complied with relevant ethical standards. An overview of the study design and analysis pipeline is outlined in Figure 9.

### 4.2. Data Sources

#### 4.2.1. Migraine Genome-Wide Association Summary Statistics

We obtained summary-level genome-wide association study (GWAS) statistics for migraine from the large-scale meta-analysis conducted by the International Headache Genetics Consortium (IHGC), as reported by Hautakangas et al. in 2022 [14]. This dataset combines four major cohorts of European ancestry (IHGC2016, UK Biobank, Manchester, UK, GeneRISK, Budapest, Hungary, and HUNT), comprising 48,975 individuals with migraine and 540,381 migraine-free controls. Migraine case status in the contributing cohorts was defined either by self-reported physician-diagnosed migraine or by meeting the International Classification of Headache Disorders criteria. Association analyses within each cohort were adjusted for age, sex, and population structure prior to meta-analysis. Due to data-sharing restrictions, individual-level and cohort-level summary statistics from the 23andMe cohort were excluded from the version of the dataset used in our study to protect participant privacy. All contributing studies obtained approval from local institutional review boards or ethics committees, and all participants provided informed consent. We analyzed only de-identified, aggregate-level summary statistics and did not access any individual-level genotype or clinical data.

#### 4.2.2. Single-Cell Transcriptomic Data

The single-cell transcriptomic data analyzed in this study were generated from an ethics-approved case–control clinical cohort. Adult participants were recruited across multiple neurology and otolaryngology centers in Spain and included individuals diagnosed with migraine as well as neurologically healthy volunteers without a history of migraine. All participants provided written informed consent prior to blood collection, and the study protocol was approved by the institutional ethics committees of the participating hospitals. Peripheral blood was drawn, and peripheral blood mononuclear cells (PBMCs) were isolated and processed under standardized conditions. Isolated cells/nuclei were encapsulated using the 10× Genomics Chromium Next GEM platform to generate single-cell RNA sequencing (scRNA-seq) and single-cell assay for transposase-accessible chromatin using sequencing (scATAC-seq) libraries. Libraries were sequenced on Illumina high-throughput platforms, and raw data were processed with the Cell Ranger/Cell Ranger ARC pipeline to produce gene-by-cell count matrices for downstream single-cell expression analysis. For downstream analyses in the present work, we used the PBMC single-cell transcriptome matrices from migraine cases and healthy controls within this cohort (GSE269117), comprising five individuals with migraine and five healthy control individuals [48].

#### 4.2.3. Healthy Human Peripheral Blood Mononuclear Cell (PBMC) Single-Cell Multi-Omics (ATAC + Gene Expression) Dataset

Using 10× Genomics Chromium single-cell multi-omics technology, PBMCs from a 25-year-old healthy female donor were analyzed. After removing granulocytes via flow cytometry sorting, cell nuclei were isolated according to the 10× Genomics standard protocol (CG000365 Rev A), yielding approximately 11,909 high-quality cells. The experiment followed the Chromium Next GEM single-cell multi-omics protocol (CG000338 Rev A) to construct paired ATAC-seq and gene expression libraries. Sequencing was performed on the Illumina NovaSeq 6000 (Illumina, San Diego, CA, USA) platform using a paired-end dual-index strategy (gene expression library cycles: 28-10-10-90; ATAC library cycles: 50-8-16-49). The final high-quality dataset included gene expression data with a median of 1826 genes and 3776 unique molecular identifier (UMI) counts per cell, as well as chromatin accessibility data with a median of 13,486 high-quality fragments per cell. A total of 108,377 open chromatin peaks and 15,494 genes were detected, with 85,468 successful peak–gene linkages, providing a reliable multi-omics dataset for investigating immune cell heterogeneity and epigenetic regulatory mechanisms.

### 4.3. Quality Control

Prior to analyzing the GWAS data, we implemented a series of quality-control (QC) steps to ensure data accuracy and reliability. The detailed methods for GWAS data QC are as follows: To ensure representativeness and reduce false positives, we first calculated the minor allele frequency (MAF) for each single-nucleotide polymorphism (SNP). By setting an MAF threshold of ≥0.01, low-frequency variants that could introduce substantial uncertainty were filtered out. SNPs below this threshold were removed to minimize bias and enhance data reliability. Due to the high linkage disequilibrium (LD) in the major histocompatibility complex (MHC) region (located on chromosome 6), which may affect GWAS analysis accuracy, SNPs in this region were removed. To ensure consistency in genome build and data format, we converted the GWAS data into a unified version and standard format for further analysis. For single-cell transcriptomic data, we applied a systematic QC workflow. First, after loading and initializing the data using the Seurat package, we calculated the percentages of mitochondrial genes (marked by “^MT-”) and hemoglobin genes (including HBA1, HBA2, HBB, etc.) to assess cell quality. Strict filtering criteria were then applied to retain high-quality cells, requiring each cell to have at least 1000 UMIs, between 200 and 5000 detected genes, a mitochondrial gene percentage ≤ 15%, and a hemoglobin gene percentage ≤ 3%. Dimensionality reduction was performed using principal component analysis (PCA), t-distributed stochastic neighbor embedding (t-SNE), and uniform manifold approximation and projection (UMAP), with quality evaluated via heatmaps and elbow plots. To eliminate batch effects from sample sources, we used the Harmony algorithm for integration correction (parameters: theta = 2, lambda = 1).

### 4.4. Tissue-Specific e/sQTL Enrichment Analysis Using QTLEnrich

Using Genotype-Tissue Expression (GTEx) v8 data across 49 tissues (splicing quantitative trait loci [sQTLs]/expression quantitative trait loci [eQTLs]), we evaluated migraine-related tissue-level associations. QTLEnrich is a rank- and permutation-based method for assessing whether phenotype associations are enriched in eQTLs and sQTLs in specific tissues, quantifying the statistical significance of these enrichments. This method accounts for three potential factors: MAF, distance to the target gene transcription start site, and local LD. Adjusted fold enrichment and enrichment *p*-value were used to evaluate the significance of the QTLEnrich method. To assess the significance and reliability of the e/sQTL enrichment analysis results in QTLEnrich, we first obtained the *p*-value for each SNP’s association with the phenotype. These *p*-values were then -log10-transformed for more intuitive visualization. Next, we plotted a quantile–quantile (QQ) plot with the x-axis as the −log10 of theoretical *p*-values (assuming that all SNPs are unrelated to the phenotype and follow a uniform distribution) and the y-axis as the −log10 of observed *p*-values.

### 4.5. Tissue-Specific Enrichment Analysis Using MAGMA

To expand on the QTLEnrich enrichment, we employed Multi-marker Analysis of GenoMic Annotation (MAGMA) enrichment analysis to explore migraine-related genomic features. First, migraine data were formatted for MAGMA. We then used the MAGMA tool for gene-level enrichment analysis, considering *p* < 0.001 indicative of credible tissues.

### 4.6. Single-Cell Spatial Transcriptomics for Migraine Tissue Enrichment Specificity

To investigate migraine, this phase integrated single-cell spatial transcriptomics (sc-ST) data with GWAS statistics to map migraine-related cellular spatial distribution patterns at single-cell resolution. We used a genetically informed spatial mapping of cells for complex traits (gsMap) algorithm, which integrates cross-species analyses of mouse embryo/brain tissues, rhesus macaque cerebral cortex, and human GWAS data to identify spatial distribution patterns of disease-related cell populations. Its core principle involves mapping trait-related genes derived from GWASs to spatially resolved cells based on their expression patterns, thereby evaluating associations between specific anatomical regions and complex traits at the cellular level. Based on the spatial transcriptomic atlas of mouse embryos at embryonic day 16.5 (E16.5; covering 25 organs), we generated migraine enrichment specificity maps along with gene spatial expression maps, establishing a single-cell resolution spatial pathogenic mechanism atlas for migraine.

### 4.7. Analysis of Migraine-Related Tissue Biological Functional Processes Using GeneEnrich

We employed gene set enrichment analysis to analyze genetic variants and expression profiles associated with migraine. This study used the GeneEnrich tool, applying hypergeometric and permutation tests to evaluate the enrichment of candidate gene sets in neurological/vascular regulation/immune-related biological functions or phenotype gene sets. To reduce tissue-specific bias, we used all detectable genes with eQTLs in each tissue as the background set, excluding genes in the MHC region, and calculated empirical *p*-values via permutation tests. This study focused on tissues and tissue proxies highly relevant to migraine pathophysiology, including peripheral and extracranial vascular-related tissues (Artery—Tibial, Artery—Aorta, and Artery—Coronary), peripheral sensory nerve proxies (Nerve—Tibial, approximating trigeminal sensory neurons/nociceptive afferent pathways), cerebral cortical regions (Brain—Cortex and Brain—Anterior cingulate cortex [BA24], reflecting cortical excitability and pain integration), and hypothalamus (Brain—Hypothalamus, reflecting autonomic/neuroendocrine-driven prodromal migraine regulation), while including Whole Blood to assess systemic immune and inflammatory contributions. The functional gene sets were sourced from Gene Ontology, Reactome, Kyoto Encyclopedia of Genes and Genomes (KEGG), Molecular Signatures Database, and Mouse Genome Informatics-related biological function databases. Statistical significance was determined as empirical *p* < 0.05 within each database for nominal significance.

### 4.8. Single-Cell Transcriptomic Atlas Annotation for Identification of Migraine Cell Signals

In this study, we conducted detailed analysis of pre-processed single-cell data, first using Harmony-corrected dimensionality reduction results for multi-dimensional visualization, including principal component heatmaps, elbow plots, and t-SNE and Harmony dimensionality reduction projection visualizations. We also evaluated cell-cycle distribution by quantifying S-phase and G2M-phase scores. Based on the Harmony dimensionality reduction results, we constructed cell neighborhoods and applied a multi-resolution strategy (13 resolutions from 0.01 to 3.0) for clustering, determining the optimal resolution of 0.01 via clustering trees to obtain stable cell subpopulations. To identify molecular features of each cell cluster, we used the FindAllMarkers function (minimum expression proportion of 25%, log-fold change threshold of 0.25) to screen cluster-specific highly expressed genes and generated heatmaps based on marker genes. Cell-type annotation was achieved using the SingleR algorithm for automated annotation.

### 4.9. Identification of Migraine Cell Signals Using ECLIPSER

Enrichment of Causal Loci and Identification of Pathogenic cells in Single Cell Expression and Regulation data (ECLIPSER) was configured with background GWAS locus scores using Bayesian Fisher’s exact test. With background GWAS locus sets as controls, we estimated cell-type-specific enrichment fold and *p*-values for each trait (GWAS locus set), tissue, and cell-type combination, where the cell-type specificity threshold was set to the 95th percentile of background locus scores. The Bayesian method estimates the 95% confidence interval (CI) for enrichment fold, suitable for traits with few loci or no loci exceeding the enrichment threshold, based on multi-level annotation data from functional genomics platforms. The specific workflow first determines the target trait and its significantly associated genetic loci based on GWAS results and then expands original signals using LD proxy relationships (r^2^ > 0.8) to comprehensively cover potential functional variants. In the preparation phase, Wilcoxon tests were used for inter-group differential expression analysis within each cell type, with key parameters: minimum cells per group = 3, minimum gene expression proportion = 10%, and log2 fold-change significance threshold = 0.5. For genes meeting differential expression criteria in specific cell types (adjusted *p* < 0.05 and absolute log2 fold-change > 0.5), ECLIPSER significance was controlled using Benjamini–Hochberg (BH) correction, with BH ≤ 0.05 cell types deemed statistically significant enriched clusters.

### 4.10. Identification of Migraine Cell Signals Using CELLECT

To assess the contribution of cell-type-specific expression to disease heritability, we used the CELL-type Expression-specific integration for Complex Traits (CELLECT) framework with two complementary methods: stratified LD score regression (S-LDSC) based on heritability and MAGMA gene analysis based on gene sets. We applied S-LDSC to analyze GWAS summary statistics for migraine. The cell-type datasets defining the analysis baseline were sourced from the tabula_muris-test and mousebrain-test datasets. LD scores were calculated using European samples from 1000 Genomes Project Phase 3 as the reference panel. *p* < 0.05 was considered significant enrichment. The MAGMA gene set analysis module tested correlations between gene-level association statistics and average gene expression levels in specific cell types. MAGMA analyzed paired combinations for given cell types to identify characteristic association signals independent of other important cell types. Similar to S-LDSC, *p* < 0.05 was considered significant enrichment.

### 4.11. Integration of Multi-Dimensional Single-Cell Evidence for Cell Prioritization

To prioritize putative disease-relevant cell types in migraine, we integrated three complementary sources: (i) a PBMC single-cell atlas (presence of annotated clusters); (ii) ECLIPSER (cell-type enrichment of GWAS signals in PBMC regulatory annotations); and (iii) CELLECT (including S-LDSC and MAGMA-based cell-type-specific expression). Each evidence source contributed one equal weight; to avoid coverage bias, non-applicable methods were flagged NA and excluded from the denominator, yielding an availability-adjusted priority index (priority index = positive/applicable). The atlas scored by presence; ECLIPSER and CELLECT scored by nominal significance (*p* < 0.05).

### 4.12. Weighted Gene Co-Expression Network Analysis in Preferred Cells to Identify Core Module Genes

Given the limited sample size (n = 10) and the lack of global heritability enrichment in the ECLIPSER analysis, we consider the following PBMC-based findings as exploratory. To describe molecular co-expression modules in the immune compartment, we performed high-dimensional weighted gene co-expression network analysis (hdWGCNA) on annotated T cells derived from the combined dataset of migraine cases and healthy controls. This pooling strategy was employed to maximize cell numbers for the construction of robust, reference-level co-expression networks independent of transient disease states. First, the annotated single-cell Seurat object was loaded, and the target cell type was set for analysis. The WGCNA function initialized the WGCNA analysis environment, selecting genes expressed in at least 5% of cells as the candidate gene set. MetacellsByGroups constructed metacells based on cell type and sample source (k = 25) to reduce single-cell data sparsity while preserving biological variation, followed by normalization and standardization of metacell expression data. After PCA dimensionality reduction, Harmony correction was employed for batch effects from sample sources. To construct the gene co-expression network, SetDatExpr extracted the expression matrix for the target cell type, and TestSoftPowers tested soft-threshold powers to select an appropriate soft threshold for building a signed topology overlap matrix network. Dynamic tree cutting identified co-expression modules and calculated module eigengenes. Further analyses examined module associations with cell types, calculated module connectivity, and renamed modules for enhanced interpretability.

### 4.13. Migraine Genomic Risk Loci Analysis

In this study, we used the Functional Mapping and Annotation (FUMA) platform for identification and annotation of genomic risk loci. First, we uploaded summary statistics files from the migraine GWAS, including SNP identifiers and genomic LD format reference values. The FUMA platform performed initial QC on these GWAS results, removing missing values and low-quality SNPs. By setting an appropriate significance threshold (*p* < 5 × 10^−8^), we screened genomic risk loci associated with migraine.

### 4.14. Conditional Analysis of Migraine Genomic Risk Loci

We performed conditional analysis on high-precision risk loci obtained from migraine risk loci to investigate whether independent secondary signals co-localized with significant GWAS signals within loci. Specifically, using the lead variant of each locus as the conditioning variable, we applied the Genome-wide Complex Trait Analysis (GCTA)-COJO tool for conditional association analysis on summary statistics. Variant allele frequencies required for analysis were sourced from the 1000 Genomes Project.

### 4.15. eCAVIAR Analysis of Migraine Genomic Loci

To identify high-confidence genes and regulatory mechanisms (eQTL/sQTL) potentially associated with migraine common risk loci, we used the Bayesian co-localization method eCAVIAR. This method evaluates whether co-occurring GWAS and e/sQTL signals tag the same causal variant or haplotype, accounting for local LD and allelic heterogeneity. eCAVIAR includes fine-mapping functionality and can handle large GWAS summary statistics without genotype data. In the analysis, we assumed up to two independent causal variants per locus. Inputs were Z-scores (beta divided by standard error) for variants from GWAS and GTEx e/sQTL studies. The LD window around each GWAS lead variant was defined as the chromosomal region containing variants with r^2^ > 0.1 (calculated using 1000 Genomes Project Phase 3 corresponding population as reference panel), extended by 50,000 bp on each side. GWAS–e/sQTL–tissue combinations with eCAVIAR co-localization posterior probability (CLPP) > 0.01 were considered significant.

### 4.16. fastENLOC Analysis of Migraine Genomic Loci

To finely identify genes and regulatory mechanisms (eQTL/sQTL) mediating associations at common risk loci for migraine, we further used the Bayesian co-localization method fastENLOC. This method uses its embedded Deterministic Approximation of Posteriors for Genetics (DAP-G) algorithm to fine-map GWAS and e/sQTLs separately, estimating posterior probabilities for each variant being causal and then assessing co-localization probability for shared causal variants without pre-setting an upper limit on independent causal variants per locus. Inputs were Z-scores (effect size divided by standard error) for variants from GWAS and GTEx e/sQTL summary data. The LD window for each GWAS lead variant was defined as the chromosomal region containing variants with r^2^ > 0.1 (using 1000 Genomes Project Phase 3 corresponding population as reference), extended by 50,000 bp on each side. We tested migraine GWAS loci against 49 GTEx tissues, with co-localization results being expressed as regional co-localization probability (RCP). As per method recommendation, GWAS–e/sQTL–tissue combinations with RCP > 0.1 were considered to have significant co-localization evidence.

### 4.17. Open Chromatin-to-Gene Expression Analysis Using Open4Gene

The purpose of open chromatin-to-gene expression analysis is to explore genes expressed in immune cells from normal tissues to determine whether stable genes in immune cells have potential associations with cells annotated in the migraine single-cell transcriptomic atlas. Open4Gene is a hurdle model-based statistical method specifically designed to handle zero-inflation common in single-cell data [49]. The analysis workflow includes the following key steps: First, scRNA-seq and scATAC-seq data were normalized, dimensionality-reduced, and clustered using Seurat and Signac packages. RNA expression matrices, ATAC peak matrices, and cell metadata (including cell-type annotations and technical covariates) were extracted from the Seurat object. ATAC peaks were then linked to gene promoter regions using a 100 kb window to define peak–gene pairs. A two-component hurdle model tested associations for each peak–gene pair: the zero component used binomial distribution (logit link) to model the relationship between ATAC openness and zero gene expression probability; the count component used truncated negative binomial distribution (log link) to model ATAC signal intensity and gene expression levels (when non-zero).

### 4.18. gsMap Gene-Level Spatial Diagnosis

After converting standardized migraine GWAS summary statistics (including variant positions, alleles, effect sizes, standard errors, *p*-values, and sample sizes) to the required trait input format for gsMap, we projected the trait onto the E16.5 mouse embryo Mouse Organogenesis Spatial Transcriptomic Atlas (MOSTA) as reference. gsMap first constructs disease-related spatial enrichment maps based on GWAS signals on the spatial atlas, then calculates consistency metrics for all detectable genes in the atlas with this spatial enrichment map, generating gene-level spatial diagnosis results. These include embryonic tissue/anatomical annotations for the gene, median gene-specificity score (Median_GSS), and Pearson correlation coefficient (PCC) between gene spatial expression and disease spatial enrichment. After analysis, genes were ranked by diagnosis scores for subsequent visualization.

### 4.19. Preferred-Gene Cell Expression Annotation and Exon Expression Analysis

Stacked bar charts and grouped UMAP were used to display cell composition proportions across samples, and specific genes (from fastENLOC analysis, eCAVIAR analysis, Open4Gene analysis, gsMap, and preferred cell weighted gene co-expression network analysis) were annotated for cell expression, including violin plots and UMAP expression projections. All analysis results were saved as high-quality image files and structured data tables. Using exon expression from the GTEx project, preferred genes were annotated for transcript types, structural features of each transcript, and expression distribution differences across human tissues.

## 5. Conclusions

In conclusion, this work presents a system-wide genetic risk atlas for migraine by integrating GWAS statistics with tissue QTLs, single-cell multi-omics, chromatin accessibility, and spatial transcriptomics. Our findings support a unifying model in which common variants contribute to migraine by perturbing several interconnected systems: (i) peripheral vascular tone and smooth muscle function in arteries, heart, and mucosa-associated organs; (ii) barrier and luminal homeostasis in mucosal epithelia, particularly in the gut and bladder; (iii) selected neuronal populations, including inhibitory cortical interneurons and peripheral sensory–autonomic neurons; and (iv) immune and inflammatory signaling pathways that intersect with vascular and neural circuits. Rather than viewing migraine solely as a disorder of brain excitability, our results reinforce that it is a multisystem neurovascular disorder with substantial peripheral input. Overall, our study illustrates the value of embedding migraine GWAS findings within a multi-layer biological framework—from organs and cell types to regulatory networks—and provides a blueprint for translating polygenic risk into experimentally testable hypotheses and, ultimately, tangible clinical advances.

## Figures and Tables

**Table 1:** Availability-adjusted prioritization of migraine-relevant cell types by integrating three evidence sources (single-cell atlas, ECLIPSER, and CELLECT; equal weight = 1 per method).

Cell Type	Single-Cell Atlas (PBMC)	ECLIPSER (PBMC)	CELLECT (S-LDSC/MAGMA)	Applicable Methods	Positive ^†^	* Priority Index *	Representative Signals (*p*)
Endothelium (mousebrain ENTG1–7)	NA	NA	1	1	1	1	ENTG3 *p* = 0.0011; ENTG2 *p* = 0.0017; ENTG4 *p* = 0.0051; ENTG1 *p* = 0.0058
Pericyte (brain, non-myeloid)	NA	NA	1	1	1	1	Tabula Muris pericyte *p* = 2.25 × 10^−4^
Vascular smooth muscle	NA	NA	1	1	1	1	VSMCA *p* = 0.0034; heart smooth muscle *p* = 1.47 × 10^−4^
Cardiac fibroblast/ myofibroblast	NA	NA	1	1	1	1	Heart myofibroblast *p* = 0.0018; Heart fibroblast *p* = 0.019
Inhibitory interneuron (TEINH18)	NA	NA	1	1	1	1	TEINH18 *p* = 0.032
Bladder epithelium	NA	NA	1	1	1	1	Bladder_bladder_cell *p* = 0.01497
Macrophage (PBMC)	1	0	NA	2	1	0.5	ECLIPSER fold enrichment = 1.51; *p* = 0.363 (BH ≈ 0.997)
T cell (PBMC)	1	0	NA	2	1	0.5	ECLIPSER *p* ≈ 0.997 (NS)
B cell (PBMC)	1	0	NA	2	1	0.5	ECLIPSER *p* ≈ 0.997 (NS)

* The Priority Index is defined as the number of positive signals divided by the number of applicable methods. Three evidence sources are weighted equally: single-cell atlas (PBMC), ECLIPSER (PBMC), and CELLECT (S-LDSC/MAGMA). Methods marked “NA” are excluded from the denominator. † Scoring is based on nominal *p*-values: Atlas (presence = 1); ECLIPSER (enrichment *p* < 0.05 = 1); CELLECT (S-LDSC or MAGMA *p* < 0.05 = 1). Abbreviations: PBMC, peripheral blood mononuclear cell; NA, not applicable; NS, not significant; S-LDSC, stratified linkage disequilibrium score regression; MAGMA, Multi-marker Analysis of GenoMic Annotation.

## Data Availability

The original contributions presented in this study are included in the article/Supplementary Material. Further inquiries can be directed to the corresponding author.

## Supplementary Materials

The following supporting information can be downloaded at: https://www.mdpi.com/article/10.3390/ijms27031615/s1.

## Abbreviations

BA24, Brodmann area 24; BH, Benjamini–Hochberg; CGRP, calcitonin gene-related peptide; CELLECT, CELL-type Expression-specific integration for Complex Traits; CI, confidence interval; CLPP, co-localization posterior probability; CSD, cortical spreading depression; DAP-G, Deterministic Approximation of Posteriors for Genetics; EBV, Epstein–Barr virus; ECLIPSER, Enrichment of Causal Loci and Identification of Pathogenic cells in Single Cell Expression and Regulation data; eQTL, expression quantitative trait locus; FDR, false discovery rate; FUMA, Functional Mapping and Annotation; GBD, Global Burden of Disease; GCTA-COJO, Genome-wide Complex Trait Analysis–conditional and joint analysis; GeneEnrich, gene set enrichment analysis tool; gsMap, genetically informed spatial mapping of cells for complex traits; GTEx, Genotype-Tissue Expression; GWAS, genome-wide association study; hdWGCNA, high-dimensional weighted gene co-expression network analysis; IBS, irritable bowel syndrome; IHGC, International Headache Genetics Consortium; KEGG, Kyoto Encyclopedia of Genes and Genomes; LD, linkage disequilibrium; MAF, minor allele frequency; MAGMA, Multi-marker Analysis of GenoMic Annotation; MHC, major histocompatibility complex; MOSTA, Mouse Organogenesis Spatial Transcriptomic Atlas; Open4Gene, open chromatin-to-gene expression integration framework; PACAP, pituitary adenylate cyclase-activating polypeptide; PBMC, peripheral blood mononuclear cell; PCA, principal component analysis; pQTL, protein quantitative trait locus; QTL, quantitative trait locus; QC, quality control; QTLEnrich, QTL Enrichment method for tissue-specific eQTL/sQTL enrichment; RCP, regional co-localization probability; scATAC-seq, single-cell assay for transposase-accessible chromatin using sequencing; scRNA-seq, single-cell RNA sequencing; S-LDSC, stratified linkage disequilibrium score regression; SNP, single-nucleotide polymorphism; sQTL, splicing quantitative trait locus; t-SNE, t-distributed stochastic neighbor embedding; TCM, central memory T cell; TCR, T-cell receptor; UMAP, uniform manifold approximation and projection; UMI, unique molecular identifier.
